# Preparation and Characterization of Polyvinyl Alcohol-Chitosan Composite Films Reinforced with Cellulose Nanofiber

**DOI:** 10.3390/ma9080644

**Published:** 2016-07-29

**Authors:** Kaiwen Choo, Yern Chee Ching, Cheng Hock Chuah, Sabariah Julai, Nai-Shang Liou

**Affiliations:** 1 Department of Mechanical Engineering, Faculty of Engineering, University of Malaya, Kuala Lumpur 50603, Malaysia; kelvin@siswa.um.edu.my (K.C.); sabsz@um.edu.my (S.J.); 2 Department of Chemistry, Faculty of Science, University of Malaya, Kuala Lumpur 50603, Malaysia; chchuah@um.edu.my; 3 Department of Mechanical Engineering, Southern Taiwan University of Science and Technology, Yungkang Dist., Tainan City 710, Taiwan; nliou@stust.edu.tw

**Keywords:** bio-nanocomposite films, polyvinyl alcohol, chitosan, cellulose, TEMPO, nanofiber, solution casting

## Abstract

In this study microcrystalline cellulose (MCC) was oxidized by 2,2,6,6-tetramethylpiperidine-1-oxyl radical (TEMPO)-mediated oxidation. The treated cellulose slurry was mechanically homogenized to form a transparent dispersion which consisted of individual cellulose nanofibers with uniform widths of 3–4 nm. Bio-nanocomposite films were then prepared from a polyvinyl alcohol (PVA)-chitosan (CS) polymeric blend with different TEMPO-oxidized cellulose nanofiber (TOCN) contents (0, 0.5, 1.0 and 1.5 wt %) via the solution casting method. The characterizations of pure PVA/CS and PVA/CS/TOCN films were performed in terms of field emission scanning electron microscopy (FESEM), tensile tests, thermogravimetric analysis (TGA), Fourier transform infrared spectroscopy (FTIR), and X-ray diffraction (XRD). The results from FESEM analysis justified that low loading levels of TOCNs were dispersed uniformly and homogeneously in the PVA-CS blend matrix. The tensile strength and thermal stability of the films were increased with the increased loading levels of TOCNs to a maximum level. The thermal study indicated a slight improvement of the thermal stability upon the reinforcement of TOCNs. As evidenced by the FTIR and XRD, PVA and CS were considered miscible and compatible owing to hydrogen bonding interaction. These analyses also revealed the good dispersion of TOCNs within the PVA/CS polymer matrix. The improved properties due to the reinforcement of TOCNs can be highly beneficial in numerous applications.

## 1. Introduction

Recently, there has been an increased interest to fabricate “green polymers” derived from natural resources in the academic and industrial areas of research [1]. Much more effort has been given to replace petroleum-derived polymers with natural, sustainable biopolymers because they are biodegradable, environmentally-friendly, and renewable with lower energy consumption [2]. Although the biopolymers displayed their potential, it is important to improve some of their properties to a certain extent that can be competitive with the petroleum derivatives, especially their poor mechanical, barrier, processing, and thermal properties [3,4,5]. 

Chitosan (CS), a natural linear polymer consisting of 1,4-linked 2-amino-deoxy-*β*-*D*-glucan, is a partially de-acetylated derivative of chitin containing the reactive amino groups. CS, the second most abundant natural polysaccharide after cellulose has unique properties, such as non-toxicity, biodegradability, renewability, and biocompatibility. CS films have been successfully used as a packaging material for protection against microbial attack and contamination in order to enhance food safety and shelf life [6]. The biopolymer is also a suitable material for biomedical applications, such as wound healing, drug delivery, tissue engineering, and numerous antimicrobial properties [7]. Polyvinyl alcohol (PVA) is a non-toxic, water-soluble, highly crystalline, biodegradable, and biocompatible polymer. It has interesting physical and chemical properties and good film-forming ability due to the abundance of hydroxyl groups and, thus, formation of intermolecular hydrogen bonding [8]. PVA is a promising semi-crystalline polymer for many applications, such as drug delivery, packaging, etc. In general, PVA is one of the synthetic polymers which is easily obtained and has a relatively low cost of production. Cellulose, one of the most abundant, renewable, and natural biopolymers, can be widely found in many forms of biomass, such as cotton, wood, and hemp, among other sources. Cellulose is a natural linear carbohydrate polymer consisting of *D*-glucopyranose units linked together by *β*-1,4-*D*-glycosidic bonds. Cellulose exists in amorphous form, but is mixed with crystalline phases through the formation of both intra- and inter-molecular hydrogen bonding and, thus, will not melt before thermal degradation [9].

The polymer blending by mixing two or more natural biopolymers (cellulose, starch, CS, chitin, etc.) and synthetic polymers (PVA, polystyrene, polylactic acid, etc.) results in the formation of new composite materials with enhanced or special properties and applications in different kinds of areas, as reported by many other researchers [10,11,12,13,14,15]. Due to environmental concerns, the composite materials should be biodegradable and recyclable, reprocessable, and reusable. In addition, the most important criteria is the sustainability and renewability of materials supplied for their production [16]. Since the blending of synthetic and natural polymers may enhance the cost performance ratio of the composite films, it is a promising strategy to blend PVA and CS to obtain the combined properties of both polymers. Despite the PVA/CS blend films providing excellent properties, a lack of flexibility is still one of the main restrictions for its application. In fact, the elongation at break of PVA/CS blend films would greatly reduce with the increase in CS content as reported by other researchers [17,18]. In addition, the thermal properties of PVA/CS blend films are still one of the obstacles due to its low thermal stability. It has been reported that the thermal stability of PVA/CS blend film would decrease with the increase in CS content [19].

Cellulose nanofibers (CNFs) or cellulose nanowhiskers (CNWs) have been gaining much more attentions in recent years because they are applicable as the natural nanofillers to produce bio-nanocomposites. There are many advantages of environmentally-friendly CNFs, such as low density, high aspect ratio, high mechanical properties, low energy consumption, biodegradability, biocompatibility, etc. Additionally, CNFs can be obtained from the abundance of renewable natural sources. However, such nanofillers have to solve many problems against industrial practices due to extremely hydrophilic surfaces, poor dispersion due to larger aggregation ability, low yield, low thermal stability, commercially unavailability, as well as relative higher price through expensive resources [20]. CNFs can be produced by 2,2,6,6-tetramethylpiperidinde-1-oxy radical (TEMPO)-mediated oxidation of celluloses, followed by mechanical disintegration of the oxidized celluloses/water [21]. TEMPO-mediated oxidized cellulose nanofibers (TOCNs), as a reinforcing phase, show high crystallinity, large aspect ratios (>50), and mostly uniform widths (3–4 nm) as compared to other nanocelluloses. Moreover, TOCNs can be homogeneously dispersed in water due to the effective electrostatic repulsion present on the anionic charge on the surfaces of TOCNs [21]. 

One of the most auspicious methods is to incorporate nanofillers into the composite blend, such as cellulose nanofibers, nanosilica, etc. [22,23,24,25,26,27,28]. The nanofillers (discontinuous phase) can be easily dispersed in a polymer matrix (continuous phase) to produce bio-nanocomposite films, where at least one dimension is less than 100 nm. In particular, some properties can be greatly improved by the use of reinforcing nanofillers even by the incorporation of only a small amount due to their large surface area. Thus, the synergetic effects of nanoreinforcements would be greatly useful for many technological and industrial applications in the future [29]. 

The aim of the present work was to produce environmental-friendly nanocellulose-based polymer composite films with enhanced mechanical, chemical, and thermal properties. More specifically, the purpose of this work was to achieve a well-dispersed nano-sized filler in the polymer matrix to improve its properties. Cellulose nanofiber was used as a reinforcing material and the combination of PVA and CS was chosen as the matrix. In order to enhance their dispersion and interfacial adhesion between the nanofiller and matrix, microcrystalline cellulose was treated by using the TEMPO-oxidation method. The oxidized microcrystalline cellulose was then mechanically converted to cellulose nanofiber. PVA/CS films were prepared at several weight ratios to evaluate the optimum behavior through some analyses. TOCN-based PVA/CS composites were solution casted at different weight compositions to produce bio-nanocomposite films. After that, various properties of the resulting films were characterized. Initially, the mechanical properties of the films were studied through the evaluation of their tensile strength (TS) and elongation at break (%E). Thermogravimetric analysis (TGA) was also carried out to study their thermal stability. The physical and chemical properties of the pure PVA/CS and PVA/CS/TOCNs films were studied through Fourier transform infrared (FTIR) spectroscopy and X-ray diffraction (XRD). Lastly, field emission scanning electron microscopy (FESEM) was conducted to investigate the effects of incorporated TOCNs content on the surface morphology of the PVA/CS films.

## 2. Results and Discussion

### 2.1. FESEM

**Figure 1:**
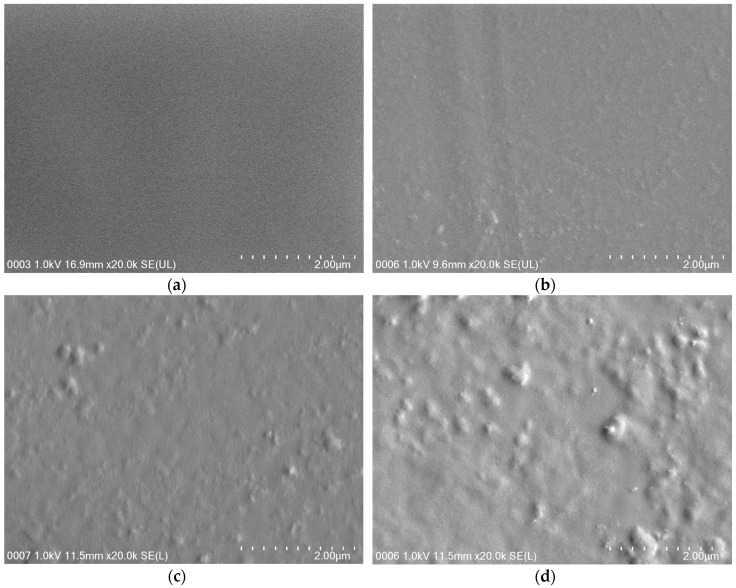
The FESEM images of the surface of PVA/CS = 50/50 films with TOCN content of (**a**) 0 wt %; (**b**) 0.5 wt %; (**c**) 1.0 wt %; and (**d**) 1.5 wt %.

Morphological tests of the films were performed using field emission scanning electron microscopy (FESEM). In general, FESEM gives information about the presence of voids, the homogeneity of the composite, the presence of aggregate, the distribution of the nanoparticles within the continuous matrix, and the possible orientation of nanoparticles [30]. The observations were performed on the surface of PVA/CS film after the synthesis. Figure 1 shows FESEM micrographs of the film surface of PVA/CS = 50/50 films with TOCN content of (a) 0 wt %; (b) 0.5 wt %; (c) 1.0 wt %; and (d) 1.5 wt %. It was observed that incorporation of TOCNs changed the microstructure of the film. The smooth surface of the blend film (Figure 1a) deduced that the homogeneous dispersion of the blend matrix. This is most likely due to formation of hydrogen bonds between the amino and hydroxyl groups of CS and the hydroxyl groups of PVA. It is difficult to observe the individual filler dispersion in the blend matrix due to its small nanoparticle size [31]. TOCNs presented as white dots in the PVA/CS films with 0.5 wt %, 1.0 wt %, and 1.5 wt % of TOCNs when compared to the PVA/CS film without the reinforcement of TOCN (control). Addition of 0.5 wt % of TOCNs gave a positive change to the microstructure (Figure 1b). A stronger interaction and adhesion between the polymer matrix and the surface of TOCNs occurred due to the homogeneous dispersion. This denser structure supported the improved tensile properties of the bio-nanocomposite films [32]. However, the surface became rougher with the addition of more TOCNs. An increase in the concentration of white dots was also observed [30]. More agglomerates were observed in the nanocomposite film with 1.5 wt % TOCNs (Figure 1d). Finally, the FESEM clarifications have allowed supporting the measured mechanical and thermal properties of bio-nanocomposite films due to the incorporation of TOCNs.

### 2.2. Tensile Properties

**Figure 2:**
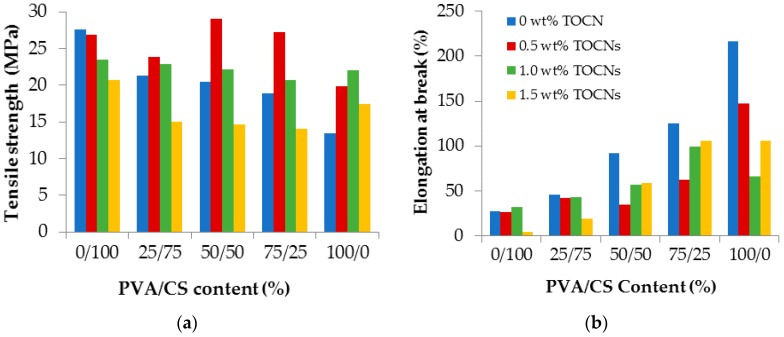
The tensile profiles in terms of (**a**) tensile strength and (**b**) elongation at break of pure PVA, pure CS, and PVA/CS films reinforced with different weight composition of TOCNs content (0, 0.5, 1.0, and 1.5 wt %).

Figure 2 shows the effects of the TOCNs content on the tensile strength (TS) and elongation at break (%E) of PVA/CS bio-nanocomposite films with different weight ratios: (a) PVA/CS = 0/100; (b) PVA/CS = 25/75; (c) PVA/CS = 50/50; (d) PVA/CS = 75/25; and (e) PVA/CS = 100/0 reinforced with different weight compositions of TOCN content (0, 0.5, 1.0, and 1.5 wt %). For the PVA/CS films without the reinforcement of TOCNs, the TS decrease with the increase of PVA contents. This could be due to more single ordered phase of PVA were formed in the matrix. In contrast, the %E of films could be increased significantly with the increase of PVA content as reported in [8]. This could be due to high molecular weight (190–310 kDa) and hard backbones of CS compared to PVA. Eventually, the addition of PVA into the CS polymer matrix could largely affect the CS polymer’s flexibility with only variations of a small change in tensile strength [33]. The TS showed the highest value when 0.5 wt % of TOCNs was added into PVA/CS = 25/75, PVA/CS = 50/50, and PVA/CS = 75/25 films. There were 11.7%, 42.1%, and 43.8% increases in TS observed when 0.5 wt % of TOCNs was added into PVA/CS = 25/75, PVA/CS = 50/50, and PVA/CS = 75/25 films, respectively. It was also observed that PVA/CS = 50/50 film with 0.5 wt % of TOCN content revealed the highest TS as compared to other PVA/CS films with 0.5 wt % of TOCN content. The reasons were likely that strong hydrogen bonding interaction between the filler and polymer blend, which enhances hard portion crystallinity, reduces motion of the molecules and, thus, increases the rigidity [32,34]. Beyond 0.5 wt %, the reduction of TS could be due to the aggregation and heterogeneous size distribution of TOCNs in the polymer matrix and, thus, the reinforcing effect of filler was inhibited. In fact, phase separation, increased formation of agglomerates, and poor particle distribution occurred due to excess TOCN content, which led to decreased tensile strength [32]. For the pure PVA, an obvious increase in TS was observed with the incorporation of TOCNs where it showed the highest value when 1.0 wt % of TOCNs were added into the polymer. This improvement could be due to the establishment of a more bonded network between PVA and TOCNs via hydrogen bonding. The relatively high strength, stiffness, and low density of TOCNs could also be the reasons for the increase of TS [35]. 

On the other hand, the addition of TOCNs reduces the %E with maximum reduction at 0.5 wt % for PVA/CS = 50/50 and PVA/CS = 75/25 films. There were 7.7%, 62.3%, and 50.5% decreases in %E for PVA/CS = 25/75, PVA/CS = 50/50, and PVA/CS = 75/25 films, respectively after addition of 0.5 wt % of TOCNs. Meanwhile, for pure PVA, the %E also decreases with the addition of TOCNs with the maximum reduction at 1.0 wt %. This reduction could be due to the stiff network structure, which strictly limited the chain mobility of the polymer matrix [32]. Such changes in the %E of composite films were reported by other researchers [12,36]. This also indicates that the blended polymers were more brittle and less flexible as compared to the pure PVA. PVA/CS = 50/50 composite was chosen for further analyses to study the effect of TOCNs on the PVA/CS films. Most importantly, it provides significant improvement in TS after the addition of 0.5 wt % of TOCNs into the PVA/CS films. Additionally, it also gives an optimum result for the test of %E.

### 2.3. TGA and DTG

**Figure 3:**
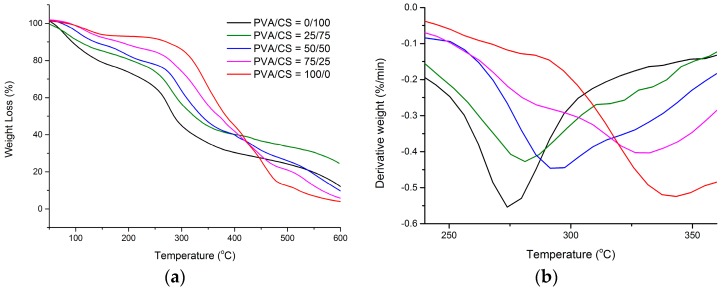
(**a**) TGA and (**b**) DTG thermograms of the PVA/CS films with different weight ratios: PVA/CS = 0/100; PVA/CS = 25/75; PVA/CS = 50/50; PVA/CS = 75/25; and PVA/CS = 100/0.

Figure 3a,b shows the TGA and DTG curves of PVA/CS blended films with different weight compositions. Table 1 gives the summary for Figure 3 in thermal parameters including T_onset_ and T_max_. It was investigated that the first weight loss appeared at about 100 °C due to the evaporation of absorbed water moisture and residual acetic acid [37]. PVA/CS = 0/100 film showed the highest weight loss, which is around 11.68%. Meanwhile, PVA/CS = 100/0 film showed the lowest loss in weight, which is only about 1.15%. Thus, it was suggested that their water-holding capacity are different in such a way that PVA/CS = 0/100 has the highest bound water content while PVA/CS = 100/0 has the lowest bound water content [38]. At 200–300 °C, a major weight loss in the bio-nanocomposite films was attributed to rapid decomposition of polymer segments of PVA and CS due to the thermal scission of the polymer backbone [8,30]. The third weight loss happened at 380–500 °C. This is caused by the degradation of the byproducts generated by PVA during its thermal degradation [8]. Generally, assuming no interaction exists between two polymers—which have different T_onset_ and, thus, the thermogram of the blends would show its thermal degradation in two different stages. However, from Figure 3, it was observed that each of the PVA/CS films show only one T_onset_ as shown on their thermograms. This indicates the presence of hydrogen bonding interactions between PVA and CS in each blend [39]. In addition, it can be noted that T_onset_ and T_max_ of the blends change slightly with the different weight composition. However, the T_onset_ and T_max_ of the blends lie between pure CS and pure PVA. These results deduced that these two polymers are well blended together [39]. From the results of the tensile test and TGA, it was observed that the thermal stability of the PVA/CS films increased with the decrease of tensile strength and increased elongation at break. Thus, it can be concluded that the tensile properties are correlated to the thermal stability of the PVA/CS composite films. 

**Figure 4:**
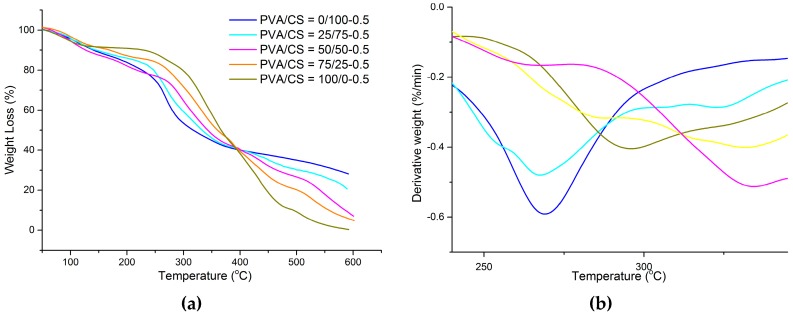
(**a**) TGA and (**b**) DTG thermograms of the PVA/CS films with different weight ratios: PVA/CS = 0/100; PVA/CS = 25/75; PVA/CS = 50/50; PVA/CS = 75/25; and PVA/CS = 100/0 at 0.5 wt % of TOCN content.

Figure 4a,b represents the TGA and DTG curves of PVA/CS films with PVA/CS = 0/100; PVA/CS = 25/75; PVA/CS = 50/50; PVA/CS = 75/25; and PVA/CS = 100/0 at 0.5 wt % of TOCN content. Table 2 gives the summary for Figure 4 of the thermal parameters of the onset temperature, T_onset_ and maximum point of the degradation, T_max_. From Figure 4a, it was observed that there are three stages of degradation. In the first stage, there were 4.36%, 4.07%, 6.41%, 3.21%, and 6.53% loss in weight for PVA/CS = 0/100; PVA/CS = 25/75; PVA/CS = 50/50; PVA/CS = 75/25; and PVA/CS = 100/0 films at 0.5 wt % of TOCN content, respectively. From the result, it showed no significant difference in the first weight loss due to evaporation of water and residual acetic acid. In the second weight loss, it can be observed that PVA/CS = 50/50-0.5 film showed the highest T_onset_ as compared to other compositions of PVA/CS films. In addition, T_max_ increases from 267 to 334 °C when PVA was added into the CS matrix. The effects of TOCNs on the degradation temperature of PVA/CS films could be due to the hydrogen bonding interactions between the –OH groups of TOCNs and the free –OH groups of PVA/CS. The strong hydrogen bonding interaction between the TOCNs and PVA/CS matrix should increase the thermal stability as the formation of a confined structure in the bio-nanocomposites [32]. From these results, PVA/CS = 50/50 film was observed to have the optimum properties from the blending of PVA and CS since it showed high thermal stability as indicated in Table 2.

**Figure 5:**
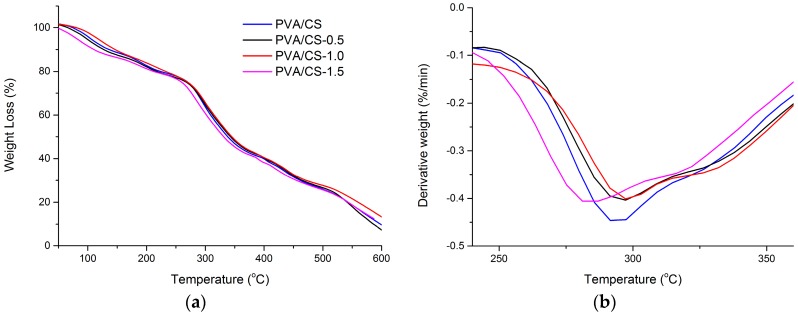
(**a**) TGA and (**b**) DTG thermograms of PVA/CS = 50/50 films with TOCN content of 0 wt %, 0.5 wt %, 1.0 wt %, and 1.5 wt %.

Figure 5a,b represents the TGA and DTG curves of PVA/CS = 50/50 films with 0 wt %, 0.5 wt %, 1.0 wt %, and 1.5 wt % of TOCN content. Table 3 gives the summary for Figure 5 in thermal parameters of onset temperature, T_onset_ and maximum point of the degradation, T_max_. From Figure 5a, there were around 4.05%, 5.37%, 2.17%, and 8.39% loss in weight observed for PVA/CS, PVA/CS-0.5, PVA/CS-1.0, and PVA/CS-1.5 films, respectively. The amount of absorbed water moisture in PVA/CS-1.0 film is the lowest as compared to other PVA/CS = 50/50 films with different TOCN content. Thus, it was suggested that the 1.0 wt % of TOCNs were well dispersed within the PVA/CS polymer matrix due to physical and molecular changes, which indicates the production of a more stable film [38,40]. From Table 3, it was indicated that T_onset_ of the pure blended film was 272 °C. After that, T_onset_ of the blended film enhanced with the increase of TOCN content until it reached the maximum of 278 °C at 1.0 wt % of TOCNs. It was noted that the difference in the T_max_ of PVA/CS films with 0 wt % and 1.0 wt % of TOCN content is only 6 °C. Thus, it can be deduced that the TOCNs content have no significant effects on the thermal stability of the films. The high thermal stability of these PVA/CS films could be due to the presence of crystalline structure and great compactness between the TOCNs and PVA/CS matrix. Thus, it can be revealed that the conversion of functional groups to –COOH groups on the TOCNs surface can significantly affect the thermal stability of the PVA/CS-based composites [40].

Upon the maximum value, the T_onset_ was then decreased to 260 °C. In fact, T_max_ of the blended films also gave a similar trend. The highest value of T_onset_ and T_max_ of PVA/CS blended films with 1.0 wt % of TOCNs indicates the improvement in the thermal stability with the addition of TOCNs. This could be due to the formation of hydrogen bonding between the –OH groups of TOCNs and –OH and –NH groups of PVA/CS films, which causes a restriction in the motion of the polymer matrix at the interfaces between PVA/CS and TOCN surfaces. In turn, the existence of hydrogen bonds should improve the value of thermal degradation due to the formation of a compact structure in the bio-nanocomposite films [32,41]. From the results of the tensile test and TGA, it was observed that the tensile strength and thermal stability of PVA/CS films increased with the reinforcement of TOCNs up to a maximum level. Both properties then decreased upon the maximum reinforcement of TOCNs. From these analyses, it can deduced that the tensile strength is correlated to thermal stability as both properties of PVA/CS films showed improvement with the reinforcement of TOCNs.

### 2.4. FTIR

**Figure 6:**
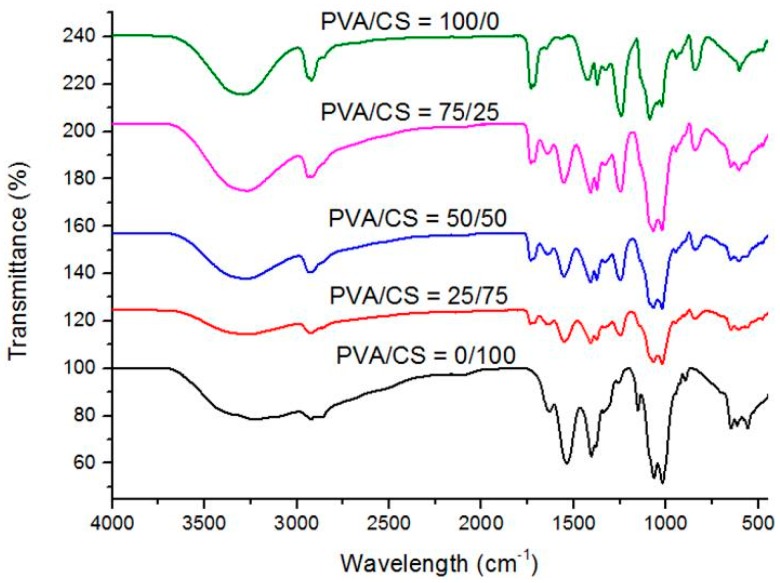
FTIR spectra of the PVA/CS films with different weight ratios: (**a**) PVA/CS = 0/100; (**b**) PVA/CS = 25/75; (**c**) PVA/CS = 50/50; (**d**) PVA/CS = 75/25; and (**e**) PVA/CS = 100/0.

Figure 6 highlights the FTIR spectra of PVA/CS blended films with different weight compositions. From the CS spectrum, the absorption band from 3450–3200 cm^−1^ is assigned to O–H and N–H stretching vibrations. The band at 2925 cm^−1^ is associated with C–H stretching. The band at 1633 cm^−1^ is attributed to C–O stretching of the acetyl group (amide I). The band at 1539 cm^−1^ is assigned to N–H bending and stretching (amide II) [31]. A weaker amino characteristic peak at 1255 cm^−1^ is associated with O–H bending vibration and the peak at 1066 cm^−1^ is assigned to C–O stretching. The absorption band at 1152 cm^−1^ and 897 cm^−1^ is assigned to the saccharine structure [42]. For pure PVA, the band at 3301 cm^−1^ is attributed to –OH stretching vibration; the peak at 1425 cm^−1^ is assigned to OH bending vibration of the hydroxyl group. The vibrational band at 2925 cm^−1^ corresponds to asymmetric CH_2_ group stretching vibration. The peak at about 1633–1561 cm^−1^ is attributed to the C=C stretching vibration of PVA. The peak corresponding to C–O stretching occurs at approximately 1089 cm^−1^ while the band at 842 cm^−1^ is attributed to the C–C stretching vibration [8,41].

From Figure 6, it was observed that a reduction in the intensity of the band at about 3301 cm^−1^ occurs with the increase in CS content in the films. This may be due to the –OH stretching vibration of PVA with secondary –NH groups of CS [8]. The increase in the PVA content in the films also caused a reduction in intensity of the band corresponding to N–H bending (amide II) at 1539 cm^−1^ of the CS film. The peak disappeared in the spectrum of the pure PVA film due to absence of the –NH functional group. In addition, an increase in the intensity of the absorption band corresponding to the C–H stretching vibration was observed at approximately 2925 cm^−1^ with the increase of PVA content. The absorption peak of the blended film at around 1245 cm^−1^ disappeared as compared to the spectrum of pure CS film [42]. Additionally, the band observed at 1066 cm^−1^ associated with the C–O stretching vibration in the spectrum of pure CS, shifted to a higher wavelength as the PVA content increases in the blend. Moreover, the intensity of the absorption band at 842 cm^−1^ corresponding to C–C stretching decreases with the increase in chitosan in the blend and, finally, the peak disappeared in the spectrum of pure chitosan film. This indicates when two or more polymers are blended together, the occurrence of physical blends and chemical interactions caused changes in the characteristic peaks of the spectra. These observations reveal the presence of good miscibility between PVA and CS. The most likely reason is the formation of intermolecular hydrogen bonds between the –OH and –NH groups in CS and the –OH groups in PVA [43].

**Figure 7:**
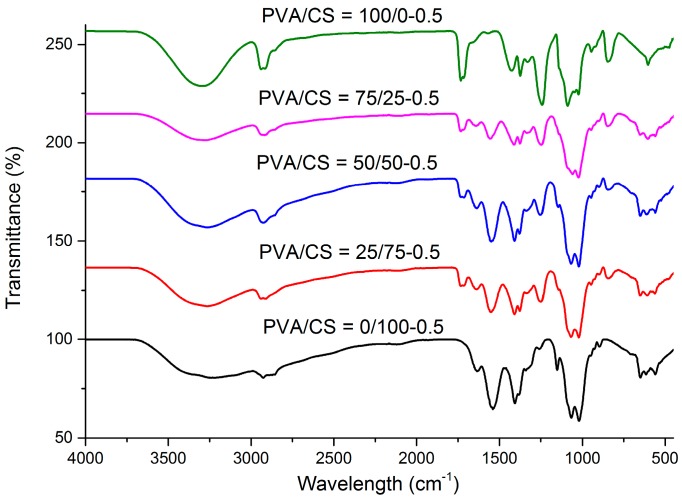
FTIR spectra of the PVA/CS films with different weight ratios: (**a**) PVA/CS = 0/100; (**b**) PVA/CS = 25/75; (**c**) PVA/CS = 50/50; (**d**) PVA/CS = 75/25; and (**e**) PVA/CS = 100/0, at 0.5 wt % TOCN content.

Figure 7 highlights the FTIR spectra of PVA/CS composite films with different weight compositions at 0.5 wt % TOCN content. At 3400–3250 cm^−1^, the intensity of the band reduces with the increase of CS content in the film. This adsorption band corresponds to the –OH stretching vibration between the PVA and CS. Additionally, the intensity of adsorption peaks at about 2927 cm^−1^ and 1245 cm^−1^ decrease with the increase of CS into the PVA matrix. This was due to the formation of hydrogen bonds between PVA and CS [42]. At about 1717 cm^−1^, the intensity of the peak decreases with the addition of CS. This peak disappears on the spectrum of PVA/CS = 0/100-0.5 film due to the absence of the C=O stretching vibration in the polymer matrix. For the characteristic peak of CS at about 1539 cm^−1^, it was also observed that intensity reduces with the increase of PVA. The peak then disappears on the spectrum of the PVA/CS = 100/0-0.5 film. This was due to the absence of –NH groups in the pure PVA film. The peak observed at 1067 cm^−1^ is associated with the C–O stretching vibration in the spectrum of the PVA/CS = 0/100 film, shifted to 1089 cm^−1^ with the increase of PVA content in the matrix. In addition, the intensity of the band at 842 cm^−1^ corresponding to C–C stretching reduces with the increase of CS content in the matrix. The peak disappears at the spectrum of PVA/CS = 0/100-0.5 film. Thus, all of the changes on the characteristic peaks revealed the good miscibility of PVA and CS in the matrix in the presence of TOCNs. It can be also deduced that there is strong hydrogen bonding interaction and interfacial adhesion between PVA/CS and TOCNs through the spectroscopic observation in Figure 7 [43]. 

**Figure 8:**
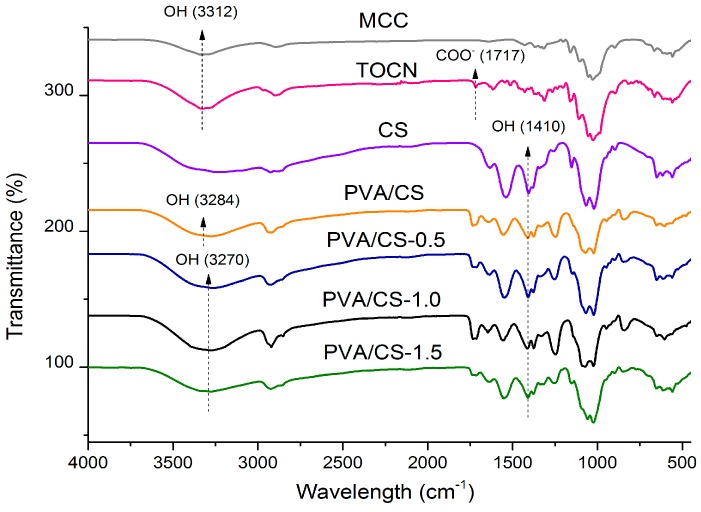
FTIR spectra of the MCC, TOCN, CS, PVA/CS = 50/50 films with TOCN content of 0 wt %; 0.5 wt %; 1.0 wt %; and 1.5 wt %.

Figure 8 shows the FTIR spectra of the MCC, TOCN, CS, and PVA/CS = 50/50 films with TOCN content of 0 wt %, 0.5 wt %, 1.0 wt %, and 1.5 wt %. From the spectrum of TOCN, the C=O stretching absorption band of sodium carboxyl and free carboxyl groups appeared as new peaks at 1614 cm^−1^ and 1717 cm^−1^, respectively, as compared to the spectrum of MCC [21]. This indicates the formation of sodium carboxyl and free carboxyl groups from the alcohol group in MCC during pH adjustment using sodium hydroxide and hydrochloric acid in the oxidation process. The C=O stretching absorption band at 1717 cm^−1^ is assigned to the C=O stretching of carboxyls with hydrogen bonds while isolated carboxyls without hydrogen bonds show a C=O absorption band at 1740 cm^−1^. It is deduced that carboxyls in the films mostly have intra- or inter-molecular hydrogen bonds with hydroxyl groups or other carboxyl groups [21]. For the PVA/CS = 50/50 film, the band observed at 3312 cm^−1^ is attributed to the –OH stretching vibration in the TOCN spectrum, shifted to approximately 3270 cm^−1^ when TOCN was added into the polymer matrix. This indicated the strong hydrogen bonding interaction between the functional group of filler and blend polymer matrix as reported in [41]. However, only minor changes are observed by the incorporation of TOCN, as expected from the low weight ratio of TOCN added to form the bio-nanocomposite films.

### 2.5. XRD

**Figure 9:**
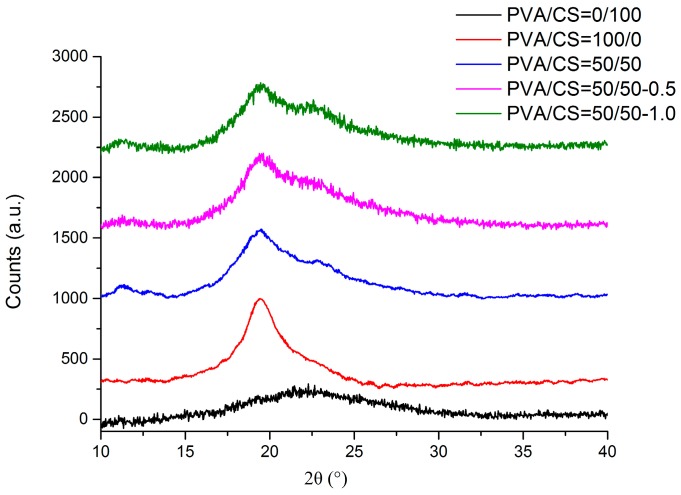
XRD data for pure PVA, pure CS, PVA/CS = 50/50, and PVA/CS = 50/50 films with TOCN content of 0.5 wt % and 1.0 wt %.

Figure 9 shows the XRD patterns of the pure CS, pure PVA, PVA/CS = 50/50, and PVA/CS = 50/50 films with 0.5 wt % and 1.0 wt % of the TOCN content. For pure CS film, the diffractogram showed three typical peaks with lower intensity at around 2θ = 11.1°, 2θ = 15.1°, and another broad peak centered at 2θ = 21.5° [44]. The peak at 2θ = 11.1° attributed to a hydrated crystalline structure and the broad peak indicated a predominant amorphous structure of CS respectively [45]. Thus, the high amorphous nature of CS film can be deduced through the broadening of the peaks [46]. For the pure PVA film, there were two peaks around 2θ = 11.0° and 2θ = 19.5° [47]. In general, if there is no interaction between two polymer components, each component would have its own crystal region in the composite. Thus, it can be deduced that the XRD patterns would be expressed as simply mixed patterns of different components in the mechanical blending case [48].

The pure PVA/CS film showed three characteristic peaks which are the crystalline phase at 2θ = 11.3° and the amorphous state with the main halo centered at 2θ = 19.4°, as well as the shoulder peak with a lower intensity at 2θ = 22.8° [49]. The diffraction peak of CS at 2θ = 15.1° disappeared in the PVA/CS = 50/50 films. For the PVA/CS = 50/50 film reinforced with 0.5 wt % of TOCNs, it indicated the three typical peaks, which are the crystalline phase at 2θ = 11.3°, the amorphous phase with the main halo of the typical peak centered at 2θ = 19.5°, and another with a lower intensity at 2θ = 23.0°. Meanwhile for PVA/CS = 50/50-1.0, the diffractogram also showed the similar trend as PVA/CS = 50/50-0.5 with the three characteristic peaks at 2θ = 11.3°, 2θ = 19.6°, and 2θ = 22.7°. As the TOCN content was increased from 0 wt % to 1.0 wt %, the peak at 2θ = 19.4° slightly increased to 2θ =19.6°. Thus, these diffractograms suggested that TOCN-reinforced PVA/CS film were composed of a combination of crystalline and amorphous peaks [50]. These results also indicate that the addition of TOCNs does not affect the uniformity in the structure of the blended polymer matrix, but rather enhance molecular ordering in the amorphous phase of the polymer matrix [32]. However, as the content of TOCNs was too low, only minor changes in wavelength or intensity are observed with the increase of TOCN content in the blended films. Lastly, XRD supported the improvement of both mechanical and thermal properties of PVA/CS films due to the reinforcement of TOCNs.

## 3. Materials and Methods

### 3.1. Materials and Chemicals

Polyvinyl alcohol (Kuraray Poval 220S, molecular weight 78 kDa, viscosity 27–33 mPa∙s, degree of hydrolysis of 87%–89%, and pH 5–7) was purchased from Kuraray Co., Ltd., Kurashiki, Japan. Cellulose, microcrystalline chitosan (molecular weight 190–310 kDa and deacetylation degree of 75%–85%), and TEMPO (98%) were purchased from Sigma-Aldrich Co. LLC., St. Louis, MO, USA. Sodium bromide (99%, AR grade) and sodium hypochlorite (10% chloride) were purchased from R & M Chemicals, Edmonton, AB, Canada. All of the chemical reagents are used without further purification.

### 3.2. Preparation of TEMPO-Mediated Oxidized Cellulose

The cellulose (12 g) was suspended in de-ionized water (575 mL) containing TEMPO (0.1946 g) and sodium bromide (1.2 g). The pH of the cellulose slurry was adjusted to 10.0 ± 0.2 with 0.5 M NaOH using a pH-meter under gentle agitation. The oxidation was started by adding the NaOCl solution (5.0 mmol NaOCl per gram of cellulose) and conducted at room temperature while stirring. The pH was maintained at 10.0 ± 0.2 by adding 0.5 M NaOH or 0.5 M HCl using a pH-meter. The reaction was quenched after 90 min by adding 30 mL of ethanol, and adjusted the pH to 7 by adding 0.5 mL HCl. The TEMPO-oxidized cellulose suspension was filtered, thoroughly washed with de-ionized water, and stored at 4 °C before further treatment or analysis.

### 3.3. Preparation of TEMPO-mediated Oxidized Cellulose Nanofiber (TOCN)

0.5% (w/v) slurry of TEMPO-mediated oxidized cellulose in de-ionized water (500 mL) was prepared and agitated at 15,000 rpm for 5 min using a mechanical homogenizer. The slurry was then sonicated for 10 min to produce TOCN with a separated dispersion using an ultrasonic bath. The disintegrated suspension was centrifuged at 10,000× *g* for 12 min to remove a small amount of unfibrillated and partially-fibrillated fractions from the supernatant containing TOCNs. The amount of TOCNs was obtained by drying three samples of 50 mL each from the supernatant at 105 °C. The suspension obtained was stored at 4 °C before further treatment.

### 3.4. Preparation of Bio-nanocomposite Films

The PVA/CS-TOCN films were prepared by the solution casting method. CS flakes were dissolved in 2.0% (w/w) aqueous acetic acid solution with continuous stirring, at 60 °C for 24 h to obtain a 1% (w/w) solution. Meanwhile, PVA was dissolved in water under constant stirring, at 80 °C to obtain a 5% (w/w) solution. Both solutions were allowed to cool until ambient temperature was reached. The TOCN solution was ultrasonicated for 20 min before continued with the blending step. The solutions obtained were blended together based on the desired mass ratios under mechanical stirring at 2000 rpm for 1 h until a homogeneous suspension is formed. Subsequently, the mixtures were transferred onto glass Petri dishes and then dried at 60 °C for 2–5 days. The dried composite films were then peeled off from their dishes, and then stored in a desiccator for future characterization use.

### 3.5. Characterization

#### 3.5.1. Morphology of Films

The surface morphology of the sample films was evaluated using field emission scanning electron microscopy (FESEM), A Hitachi SU8220 (Tokyo, Japan) was used with an operating voltage of 1.0 kV at a magnification of 20,000× at room temperature. Each sample was put on a holder before being coated with a thin platinum layer to avoid the charging effect.

#### 3.5.2. Tensile Properties of Films

The tensile strength (TS) and elongation at break (Eb) of the films were measured as per ASTM D 882 test methods, using an Autograph AGS-X Universal Tester (Shimadzu, Kyoto, Japan). The tensile samples were cut into rectangular shapes with dimensions of 100 mm in length and 10 mm in width. The gauge length was fixed at 50 mm and the speed of the moving clamp was 5 mm min^−1^. Five samples were tested and the average values were taken as the reported results.

#### 3.5.3. TGA Analysis of Films

The thermogravimetric analysis (TGA) of the films was conducted using a Mettler Toledo TGA/SDTA851 thermogravimeter (Mettler Toledo Coro, Greifensee, Switzerland). The sample size was approximately 10 mg. The samples were heated at the rate of 15 °C·min^−1^ from 35 to 600 °C under flowing air.

#### 3.5.4. FTIR Analysis of Films

The Fourier transform infrared spectroscopy (FTIR) analysis of the sample films was performed using a FTIR Spectrum 400 (Perkin Elmer, Waltham, MA, USA). The analysis was carried out in the range from 4000 to 400 cm^−1^ with a 4 cm^−1^ resolution and a total of 32 scans. The FTIR spectra were recorded in transmittance mode. 

#### 3.5.5. XRD Analysis of Films

The X-ray diffraction (XRD) analysis of the films was carried out using a Rigaku (Tokyo, Japan) X-ray diffractometer. The instrument was operated at 40 kV and 40 mA and the X-ray radiation was nickel-filtered Cu (wavelength = 0.1542 nm). The samples were analyzed over a scanning scope of 2θ from 5° to 80° with a step increment of 0.02°/s at room temperature.

## 4. Conclusions

In this current study, there are few conclusions that can be deduced after completely performing the characterization tests. In summary, cellulose nanofiber-reinforced PVA/CS bio-nanocomposites with various amounts of TOCNs were prepared through a solution casting method and then followed by characterization tests. The observation of the surface morphology of the bio-nanocomposite film showed that the TOCNs were homogeneously dispersed at low filler loading and started to agglomerate at 1 wt % of TOCNs. The tensile profile indicated that the tensile strength of PVA/CS composite films at low TOCN loading was stronger than those films without the reinforcement of the filler. In contrast, the flexibility of PVA/CS composite films was reduced at low filler loading. From the thermal study, the TOCNs have only caused slight changes to the thermal stability of PVA/CS composite films. As evidenced by the structural characterization by FTIR and XRD analyses, both the PVA and CS polymers proved to be compatible and homogeneously mixed together via the interfacial adhesion and hydrogen bonding interaction. These analyses also indicated the presence of the strong interaction between the TOCNs and the PVA/CS polymer matrix which led to better dispersion of the nanofiller within the polymer matrix. In conclusion, the current outcomes will give an advantageous insight of developing biodegradable and renewable bio-nanocomposite films that will be highly useful for a wide range of applications.

## Figures and Tables

**Table 1:** Summary of TGA and DTG thermograms of the PVA/CS films with different weight ratios: PVA/CS = 0/100; PVA/CS = 25/75; PVA/CS = 50/50; PVA/CS = 75/25; and PVA/CS = 100/0 in terms of onset temperature, T_onset_ and maximum temperature of the degradation, T_max_.

Sample	First Step	
	T_onset_ (±5 °C)	T_max_ (±5 °C)
PVA/CS = 0/100	261	274
PVA/CS = 25/75	267	281
PVA/CS = 50/50	272	293
PVA/CS = 75/25	278	329
PVA/CS = 100/0	287	340

**Table 2:** Summary of TGA and DTG thermograms of the PVA/CS films with different weight ratios: PVA/CS = 0/100; PVA/CS = 25/75; PVA/CS = 50/50; PVA/CS = 75/25; and PVA/CS = 100/0 at 0.5 wt % of TOCN content in terms of onset temperature, T_onset_ and maximum temperature of the degradation, T_max_.

Sample	TOCNs (wt %)	First Step	
		T_onset_ (±5 °C)	T_max_ (±5 °C)
PVA/CS = 0/100	0.5	241	267
PVA/CS = 25/75	0.5	243	268
PVA/CS = 50/50	0.5	273	296
PVA/CS = 75/25	0.5	260	332
PVA/CS = 100/0	0.5	253	334

**Table 3:** Summary of TGA and DTG thermograms of PVA/CS = 50/50 films with TOCN content of 0 wt %, 0.5 wt %, 1.0 wt %, and 1.5 wt % in terms of onset temperature, T_onset_ and maximum temperature of the degradation, T_max_.

Sample	TOCNs (wt %)	First Step	
		T_onset_ (±5 °C)	T_max_ (±5 °C)
PVA/CS	0	272	293
PVA/CS/TOCNs	0.5	273	296
PVA/CS/TOCNs	1.0	276	299
PVA/CS/TOCNs	1.5	260	284

## Abbreviations

The following abbreviations are used in this manuscript:

CNF: Cellulose nanofiber
CNW: Cellulose nanowhisker
CS: Chitosan
DTG: Derivative thermogravimetic analysis
%E: Elongation at break
FESEM: Field emission scanning electron microscopy
FTIR: ourier transform infra-red
MCC: Microcrystalline cellulose
PVA: Polyvinyl alcohol
T_max_: Maximum point of degradation
T_onset_: Onset temperature
TGA: Thermogravimetric analysis
TOCN: TEMPO-mediated oxidized cellulose nanofiber
TS: Tensile strength
XRD: X-ray diffraction
